# Changes in Physicochemical Properties and Volatiles of Kiwifruit Pulp Beverage Treated with High Hydrostatic Pressure

**DOI:** 10.3390/foods9040485

**Published:** 2020-04-12

**Authors:** Yajing Chen, Xiaoping Feng, Hong Ren, Hongkai Yang, Ye Liu, Zhenpeng Gao, Fangyu Long

**Affiliations:** 1 College of Food Science and Engineering, Northwest A&F University, Yangling 712100, China; jeanchen@nwafu.edu.cn (Y.C.); xiaoping-feng@nwafu.edu.cn (X.F.); renhong624@outlook.com (H.R.); hongkai1154@163.com (H.Y.); gzp5988@163.com (Z.G.); 2 China-Canada Joint Lab of Food Nutrition and Health (Beijing), Beijing Technology & Business University, Beijing 100048, China; liuye@th.btbu.edu.cn

**Keywords:** high hydrostatic pressure, kiwifruit pulp beverage, storage, physicochemical properties, volatiles

## Abstract

Physicochemical properties and volatiles of kiwifruit pulp beverage treated with high hydrostatic pressure (HHP, 400–600 MPa/5–15 min) were investigated during 40-day refrigerated storage. Compared with heat treatment (HT), HHP ranged from 400–500 MPa was superior in retaining vitamin C, fresh-like color and volatiles, while soluble solids content and pH were not affected significantly. Furthermore, HHP improved brightness and inhibited browning of kiwifruit pulp beverage. Samples treated at 400 MPa for 15 min showed significantly higher vitamin C content and lower ∆E values over 40 days than heat-treated kiwifruit pulp beverage. The total content of alcohols, esters, acids, and ketones gradually increased, whereas the total aldehydes content decreased during storage. Interestingly, HHP treatment at 500 MPa for 15 min mostly retained important characteristic volatiles including hexanal and *(E)*-2-hexenal, indicating this treatment was more conducive to preserve the original fruity, fresh, grassy and green notes of kiwifruit pulp beverage than HT.

## 1. Introduction

Kiwifruit is famous as the king of fruits due to unique flavor and high content of nutrients including minerals, soluble dietary fiber, and vitamin C, the content of which in kiwifruit is 3–5 times more than that in citrus fruits [1,2]. Furthermore, kiwifruit contains folic acid, lutein, natural inositol, carotene, and other nutrients [3,4]. It has been confirmed that kiwifruit has many potential health benefits such as anticancer, inhibiting tumor cells, decreasing cholesterol, and promoting heart health [5]. However, kiwifruit is a respiration climacteric fruit, which rot easily and get soft after harvesting. As a consequence, it is necessary that kiwifruit should be further processed and sterilized to get longer shelf life.

To date, the post-harvest preservation methods of kiwifruit mainly include low-temperature refrigeration, modified atmosphere storage, 1-methylcyclopropene (1-MCP) treatment, and ozone treatment [6,7]. Despite the possibility that these techniques could delay the maturity and senescence of kiwifruit, promoting the use of the modified atmosphere storage, cold-chain transportation, and 1-MCP widely is difficult because of the limitations of equipment and technology. Besides, fruits need to be treated with ozone several times during storage, which is very complicated. High concentration of ozone will also accelerate the spoilage of kiwifruit and even cause harm to human. In addition to the above preservation methods, the shelf life of kiwifruit can also be extended by further processing. Kiwifruit products such as kiwifruit juice, fruit wine and kiwifruit jam have attracted great attention worldwide owing to their delicious taste and convenience [8,9]. However, the processing method of kiwifruit products available on the market nowadays mostly is heat treatment, which is easy to operate and at low cost. Meanwhile, heat treatment usually causes serious lost in vitamin C, changes in color, adverse effects on sensory and reduction of volatiles [10,11]. Given that consumers desire for higher quality and fresher products, it is important to seek a sterilization technology that retains the aroma and nutrients of products as much as possible.

As a non-thermal sterilization technique, high hydrostatic pressure (HHP) inactivates microorganisms in food by pressure ranges from 100 MPa to 1000 MPa at room temperature, thereby extending the shelf life of food [12,13]. Compared with traditional heat treatment (HT), HHP exerts much less impact on the original aroma, freshness color and vitamins of food [14,15]. The study of Wang et al. [16] suggested that −a* value of HHP-treated spinach puree was higher than that of heat-treated samples during storage, which revealed that HHP could delay the deterioration of green color in spinach puree. Moreover, it has been observed that vitamin C of litchi based mixed fruit beverage was preserved after HHP processing at 400 MPa/600 MPa and 30 °C for 20 min [17].

Effect of HHP on volatiles in fruits has also been evaluated. Study by Fernandez Garcia et al. [18] showed that HHP processing (800 MPa for 5 min) intensified carrot aroma of fresh orange-lemon-carrot juice. Krebbers et al. [19] also observed that the content of methyl chavicol and linalool, which are important to flavor of fresh basil, were unchanged after HHP treatment at 860 MPa for 75 °C as well as 700 MPa for 85 °C. According to previous studies, it has been confirmed that *(E)*-2-hexenal and hexanal are main characteristic volatiles which contribute to the grassy and fruity notes in kiwifruit [20]. Hexanal imparts grassy and leafy notes to kiwifruit. *(E)*-2-hexenal is responsible for green and fruity aroma [21]. Apart from these two main volatiles, several volatiles also affect flavor of kiwifruit. For instance, ethyl acetate is an odorant with fruit and pineapple aroma while 1-hexanol provides grassy, floral, and soil notes to kiwifruit. Besides, butyl acetate relates to sweet of kiwifruit [22]. Most studies concentrated on investigating changes of aroma in kiwifruit during storage and ripening. Wan et al. [23] observed an increasing trend with 1-hexanol during storage. Besides, volatiles of kiwifruit were very sensitive to storage time and the total volatiles content enhanced with increasing storage time. It is also reported that levels of esters tended to enhance, while aldehydes which give kiwifruit green-smelling decreased gradually during ripening [24]. The main focus of studies was on the positive effects of HHP on preserving pH, vitamin C content and color of kiwifruit. Nevertheless, the effect of HHP on volatiles in kiwifruit has been evaluated in limited research. Moreover, it remains unclear what changes of volatiles in HHP-treated kiwifruit during storage.

The objective of this study was to assess how HHP treatment affects the volatiles in kiwifruit pulp beverage during 40-day storage at 4 °C. In addition, the impacts of HHP treatment on pH, soluble solid content, vitamin C content, and color of kiwifruit pulp beverage during storage were also investigated.

## 2. Materials and methods

### 2.1. Materials and Reagents

Fresh kiwifruits were purchased from a local market of Yangling (Shaanxi, China). The salt, citric acid, ascorbic acid and sugar were all food grade and were purchased from Guangzhou Fuzheng Donghai Food Co., Ltd (Guangzhou, China). The oxalic acid, sodium bicarbonate, 2,6-dichlorophenol sodium salt and L(+)-ascorbic acid were analytical grade and were purchased from Sichuan Xilong Chemical Co., Ltd. (Chengdu, China). The pectinase was gained from the Sigma-Aldrich, St. Louis (MO, USA).

### 2.2. Preparation of Kiwifruit Pulp Beverage

After peeling, washing and cutting, kiwifruits were soaked in the color-protection solution (1:3, *w/v*) for 30 min, which consisted of 1% (*w/v*) salt, 0.15% (*w/v*) citric acid and 0.05% (*w/v*) ascorbic acid. Then kiwifruits were juiced to kiwifruit pulp. Pectinase was used to hydrolyze pectin in kiwifruit at concentration of 450 mg/L for 1 h. Thereafter, kiwifruit pulp was diluted with ten volumes of distilled water, 100 g/L sugar and 1 g/L citric acid were added into pulp beverage followed. After that, the samples were vacuum sealed and stored at 4 °C before HHP treatment.

### 2.3. HHP and Heat Treatments of Kiwifruit Pulp Beverage

The kiwifruit pulp beverage samples were placed into a hydrostatic pressurization unit (SHPP-8.8L, Shanxi Sanshuihe Technology Co., Ltd., Shanxi, China) at room temperature and processed using high pressure levels from 400 to 600 MPa and pressure-holding time from 5 to 15 min, respectively. Heat treatment was carried out by heating samples in a water bath at 85 °C for 10 min.

### 2.4. Storage Conditions

Kiwifruit pulp beverage samples after HHP and heat treatments were stored in the dark at 4 °C for 40 days. Pulp beverage samples were analyzed after 0, 10, 20, and 40 days.

### 2.5. Determination of pH and Soluble Solids Content (SSC)

pH value was detected with a pH meter (PHS-3C, Shanghai Leici Instrument Co., Ltd., Shanghai, China). SSC was detected with a hand-held refractometer and was expressed as °Brix.

### 2.6. Assessment of Vitamin C (VC) Content

The determination of vitamin C content followed the method in study of Xu et al. [6] with modifications. Kiwifruit pulp beverage (20 mL) was transferred into a 100 mL volumetric flask and diluted to the scale with oxalic acid (2%). The mixture was filtered after blending. Ten milliliters of filtrate was then titrated with 2,6-dichloroindophenol until the solution turned pink and kept for 15 s. The equation for calculating vitamin C content was as follows:

Vitamin C content (mg/100g) = ((V − V_0_) × T × A)/V_K_) × 100 (1)

where V and V_0_ represents the volume of 2,6-dichloroindophenol used in titration of the sample and white respectively, A indicates the dilution ratio of the samples, V_K_ represents the volume of the samples which is drawn, T refers to the titer of the 2,6-dechloro-indophenol solution, C is the mass concentration of the ascorbic acid standard solution (1.000 mg/mL), V_1_ represents the volume of the ascorbic acid solution (1 mL) and V_c_ is the volume of solution used in titration of ascorbic acid.

T = (C × V_1_)/(V_c_ − V_0_) (2)

### 2.7. Color Assay

The changes in color of kiwifruit pulp beverage were measured in transmission mode using a colorimeter (CI-7600, X-RITE, Grand Rapids, MI, USA). The readings obtained by the measurement are L*, a* and b* values. L* value indicates brightness, a* value represents greenness (−) or redness (+) and b* value represents blueness (−) or yellowness (+) of samples. The total color difference (∆E) was determined according to the following equations where ∆L*, ∆a*, ∆b* were the differences between the values of treated samples and those of untreated samples at the initial time.

(3) $$\Delta E=\sqrt{\Delta L^{*2}+\Delta a^{*2}+\Delta b^{*2}}$$

### 2.8. Analysis of Volatiles

Determination of volatiles was performed by solid phase micro-extraction (SPME) and gas chromatography-mass spectrometry (GC-MS, QP 2010 ULTRA, Shimadzu Corporation, Kyoto, Japan). The method of collecting the volatiles was according to the previous study of Zheng et al. [25] with modifications. Five milliliters of kiwifruit pulp beverage was added into closed sample vials which contained 1.5 g NaCl. A solid-phase micro-extraction needle (50/30 µm DVB/CAR/PDMS, Sigma-Aldrich, St. Louis, MO, USA) with a 1 cm long fiber was used for volatile extraction. The extraction time lasted 30 min. During extraction, the sample was stirred continuously at 750 rpm using a magnetic stirrer. After extraction, SPME fiber was introduced into the GC-MS splitless injector and maintained at 250 °C for 3 min for desorption of samples [24]. The gas chromatograph was equipped with a DB-17MS capillary column and He was the carrier gas of which the rate was 1.93 mL/min. For analyzing the volatiles, the GC oven temperature remained at 40 °C for 3 min, raised to 240 °C at 4 °C/min and then kept for 9 min. The injection port temperature was 250 °C. The measurement was performed by electron ionization mass spectrometry at 70 eV and a scanning range of 35–500 *m/z*. The interface temperature and ion source temperature were both 230 °C. Volatiles were identified based on retention time and matching degree by comparing experimental mass spectrometry data with NIST14 database. The concentration of each volatile was evaluated through the peak area.

### 2.9. Statistical Analysis

All treatments were carried out in triplicate for each condition and all measurements were done in triplicate. All data were expressed as means ± standard deviation (SD). The significant differences on the results were analyzed by one-way analysis of variance (ANOVA) followed Fisher’s least significant difference (LSD) test using SPSS software (20.0, Chicago, IL, USA). The mean values were considered to be different significantly at *p* < 0.05.

## 3. Results and Discussion

### 3.1. Changes in Soluble Solids Content (SSC) and pH of HHP-Treated Kiwifruit Pulp Beverage

Compared to untreated samples, there were no significant changes of pH and SSC after HHP treatments (The minimum and maximum soluble solids content obtained for kiwifruit pulp beverage were 10.5 ± 0.41 °Brix and 11.0 ± 0.00 °Brix, respectively). These results were in agreement with study of Zhang et al. [26] who reported that pH in carrot juice did not show significant change after HHP and heat treatments. Chen et al. [27] also found that SSC of cloudy pomegranate juice after HHP treatment had a slight fluctuation during storage. Besides, no significant variations of SSC were observed in kiwifruit pulp beverage during 40-day storage. Although there was an increase (*p* < 0.05) in pH from the first day to the 10th day, pH remained stable until the end of storage period (Table 1). Therefore, pH of kiwifruit pulp beverage was not changed significantly from day 10 to day 40. Similarly, Cortes et al. [28] found that pH of orange-carrot juice increased significantly during storage. However, studies by Alighourchi et al. [29] and Bull et al. [30] found no significant change during 210-day storage and 12 week-storage in pH of pomegranate juice and the navel orange juice, respectively. Besides, Barba et al. [31] observed that HHP treatment at 100–400 MPa for 2–9 min and at 20–42 °C caused no statistically significant change on SSC of vegetable beverage.

### 3.2. Effect of HHP on Vitamin C Content in Kiwifruit Pulp Beverage

**Figure 1:**
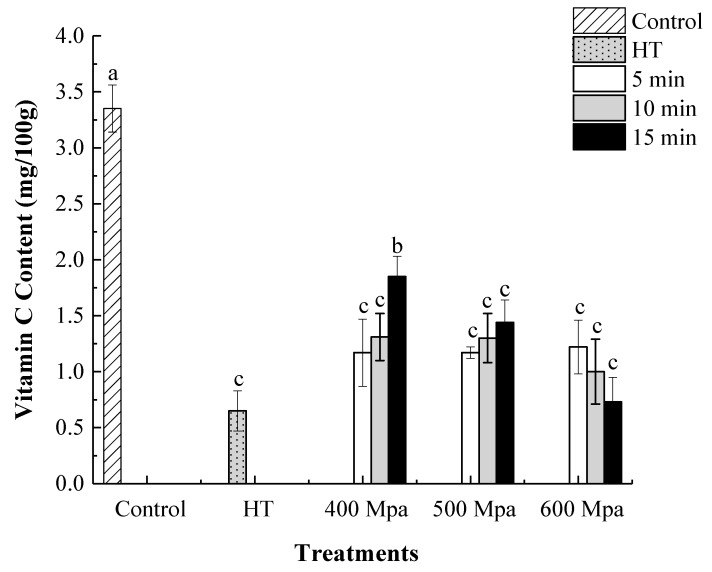
Vitamin C content of HHP-treated and heat-treated kiwifruit pulp beverage after 0 day. Control (kiwifruit pulp beverage treated without heat treatment (HT) or HHP). HT (85 °C for 10 min), HHP (high hydrostatic pressure, 400–600 MPa for 5–15 min). Values with different letters are significantly different (*p* < 0.05). All data were expressed as means ± SD, *n* = 3.

As an essential nutrient component in kiwifruit, vitamin C is not stable and always decomposed and oxidized when exposed to light and oxygen. Consequently, VC content is considered as an important index of oxidative deterioration in fruit juices [32]. As can be seen in Figure 1, HHP and heat treatments caused a pronounced reduction in VC content (*p* < 0.05). Interestingly, samples processed at 400 MPa for 15 min showed the highest VC content with 35.8% higher VC retention compared to HT. Similarly, Castro et al. [33] found that HHP-treated green bell peppers showed a higher vitamin C retention than the samples treated by thermal blanching at 80 °C and 98 °C.

**Figure 2:**
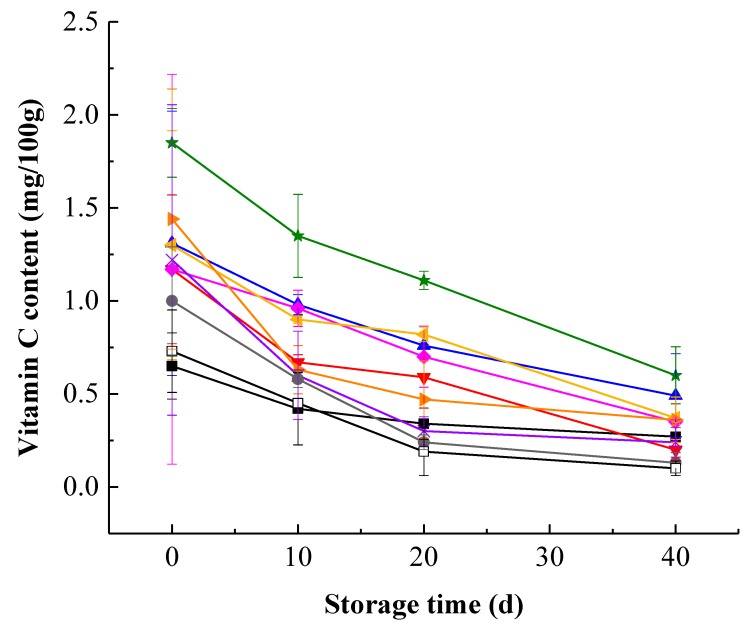
Changes of vitamin C content in heat-treated and HHP-treated kiwifruit pulp beverage during storage at 4 °C for 40 days. Heat treatment (85 °C for 10 min) (■), HHP (high hydrostatic pressure, 400–600 MPa for 5–15 min): 400 MPa/5 min (**▼**), 400 MPa/10 min (**▲**), 400 MPa/15 min (**★**), 500 MPa/5 min (**◆**), 500 MPa/10 min (**◀**), 500 MPa/15 min (**▶**), 600 MPa/5 min (**×**), 600 MPa/10 min (**●**), 600 MPa/15 min (□). All data were expressed as means ± SD, *n* = 3.

Kiwifruit pulp beverage showed a gradual decrease in VC content during storage, proving that vitamin C degraded with time (Figure 2). This is in line with the result of Xu et al. [34] who observed a significant reduction in VC content when the storage time progressed. Moreover, the degradation rate of vitamin C in the early stage of storage was greater than that in the later stage of storage, which may be due to a decrease in oxygen concentration during storage, thus the degradation of vitamin C gradually changed from aerobic degradation to anaerobic degradation, while the rate of aerobic degradation is greater [35]. During the whole storage period, HHP treatment at 400 MPa for 15 min maintained significantly (*p* < 0.05) higher VC content and showed better effect on maintenance of vitamin C. This suggested that HHP treatment at 400 MPa for 15 min protected vitamin C of kiwifruit pulp beverage against long-term degrading processes. Nevertheless, with higher HHP of 600 MPa for 15 min, the VC content of samples decreased sharply and was even lower than that in heat-treated samples, which revealed that extremely high pressure could cause more loss in vitamin C. Similar results were obtained by Landl et al. [36] who treated acidified Granny Smith apple puree at 600 MPa, found the total vitamin C content fell significantly.

### 3.3. Changes in Color of HHP-Treated Kiwifruit Pulp Beverage

The color parameters of kiwifruit pulp beverage treated by HHP and heat treatments are presented in Table 2. L* values increased slightly as the pressure increased. Lightness and yellowness of kiwifruit pulp beverage were not affected by HHP and heat treatments since no significant change was found in L* value and b* value after treatments (*p* > 0.05). Xu et al. [37] reported that HHP at 550 MPa for 5 min showed no effect on b* and L* values of pepper and orange juice blend, which was similar to our results. Furthermore, all treatments had no significant effect on a* values in addition to HT and treatment at 600 MPa for 10 min, indicating that HT and extremely high pressure would have significant impacts on greenness. ΔE value refers to the total color difference. In this study, ΔE values tended to increase with increasing pressure. HHP-treated pulp beverage showed lower ΔE values than heat-treated pulp beverage, which illustrated that HHP maintained the original color of samples better than HT. Previous studies also reported that ΔE values of apricot nectars and mango nectar treated by high temperature short time were higher than ΔE values of HHP-treated samples [38,39]. Especially, ∆E value of samples processed at 400 MPa for 15 min was found to be significantly lower than those of the other treatments and thence provided better conservation of original color.

**Figure 3:**
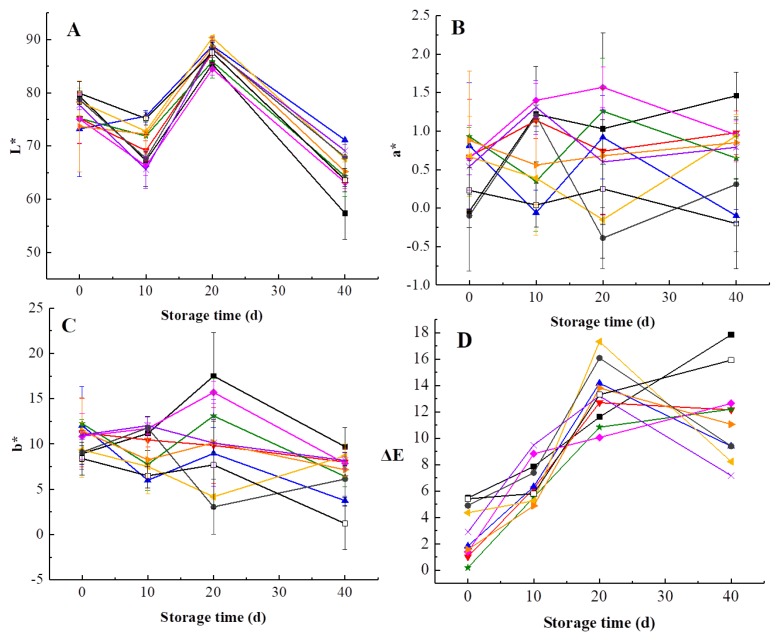
Changes in color of heat-treated and HHP-treated kiwifruit pulp beverage during storage at 4 °C for 40 days. (**A**) L*, the brightness value on a black (0) to white (100) scale; (**B**) a*, hue on a green (−) to red (+) scale; (**C**) b*, hue on a blue (−) to yellow (+) axis; (**D**) ∆E, the total color difference between treated samples and untreated samples at the initial time. Heat treatment (85 °C for 10 min) (■), HHP (high hydrostatic pressure, 400–600 MPa for 5–15 min): 400 MPa/5 min (**▼**), 400 MPa/10 min (**▲**), 400 MPa/15 min (**★**), 500 MPa/5 min (**◆**), 500 MPa/10 min (**◀**), 500 MPa/15 min (**▶**), 600 MPa/5 min (**×**), 600 MPa/10 min (**●**), 600 MPa/15 min (□). All data were expressed as means ± SD, *n* = 3.

Kiwifruit is likely to undergo browning during storage, which affects the sensory quality of products. As can be observed in Figure 3, L*, a*, b*, and ΔE values of kiwifruit pulp beverage changed during 40-day storage. L* value of heat-treated samples changed significantly and the lowest L* value was obtained at day 40 for HT, which was lower than L* values of pulp beverage treated by HHP, illustrating the maintaining effect of HHP in brightness is better than HT with the prolongation of storage period. L* value increased after 20 days of storage, which may be related to decrease of VC content and total polyphenol [34]. Additionally, a* values increased gradually during storage, stating that a gradual loss of green in all treated samples. As shown in Figure 3C, b* values increased first and then reduced after 20 days. HHP-treated samples remained lower b* values in comparison to heat-treated samples during storage, which meant HHP inhibited browning of pulp beverage compared with HT.

There was an increase trend in ΔE values of kiwifruit pulp beverage over 40 days (Figure 3D). The result was in agreement with previous studies about clear cucumber juice and purple sweet potato nectar, which concluded that ΔE values increased during storage [40,41]. It is obvious that the difference of ∆E values between different treatments gradually increased with the storage time. Besides, ΔE values of heat-treated samples as well as samples treated at 600 MPa/15 min increased sharply, illustrating that HT, extremely high pressure and long treatment time would significantly change the color of kiwifruit pulp beverage. On the contrary, HHP ranged from 400 MPa to 500 MPa exerted protective effects on color of pulp beverage. Particularly, the significantly lower ΔE values in pulp beverage treated at 400 MPa for 15 min during 40-day storage time clearly demonstrated this treatment could retain the fresh-like color and appearance of kiwifruit pulp beverage. A similar trend was also observed in strawberry and blackberry purees, Patras et al. [42] reported that ΔE values of puree treated by HHP were smaller than that of thermal treated samples.

### 3.4. Volatiles Analysis

#### 3.4.1. Changes of Volatiles in Kiwifruit Pulp Beverage after HHP

**Figure 4:**
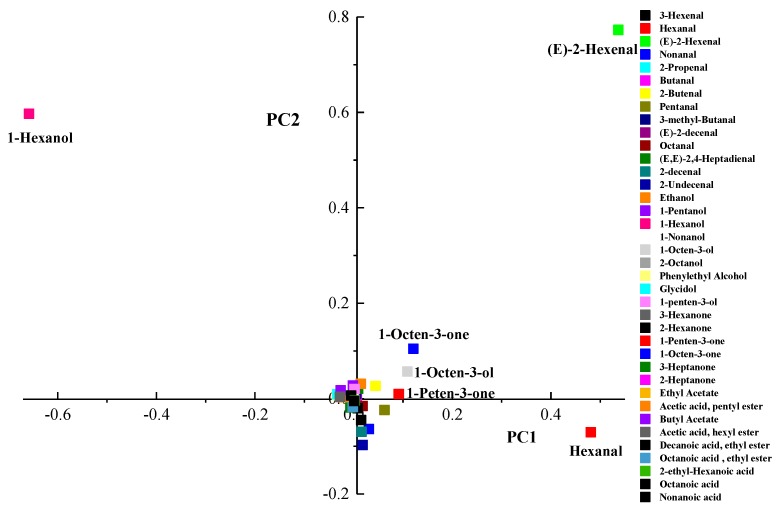
Loading plots after principal components analysis of the variables in the plane defined by the two first principal components (PC 1 and PC 2).

**Figure 5:**
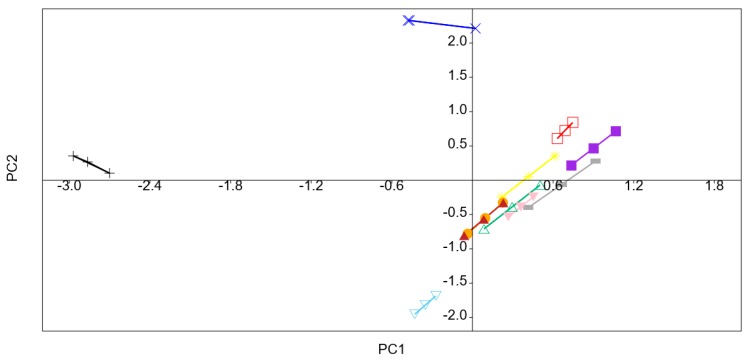
Principal component analysis (PCA) plot of the volatiles of kiwifruit pulp beverage at different treatment conditions. Untreated (+); HT (85 °C for 10 min) (**□**); 400 MPa/5 min (**■**); 400 MPa/10 min (**×**); 400 MPa/15 min (※); 500 MPa/5 min (**△**); 500 MPa/10 min (**—**); 500 MPa/15 min (**▼**); 600 MPa/5 min (**●**); 600 MPa/10 min (**▲**); 600 MPa/15 min (**▽**).

Despite high pressure did not directly change the fresh flavor of fruits and vegetables, HHP could change the concentration of volatiles indirectly because of enhancing or slowing down some enzymatic and chemical reactions [43]. A principal component analysis (PCA) was carried out to evaluate effect of different treatments on volatiles of kiwifruit pulp beverage. Figure 4 showed the score plot of the different variables (coefficients of the eigenvectors) for the two first principal components (PC 1 and PC 2). The most important contributions to PC1 were hexanal, *(E)*-2-hexenal, 1-hexanol, 1-octen-3-ol, 1-octen-3-one, and 1-penten-3-one since they were far from the origin and explained an important part of the variation. The first principal component (PC) explained 69.2% and the second PC explained 20.9%, altogether 90.1% of the variation among the samples. As can be seen in Figure 5, volatiles of kiwifruit pulp beverage were modified by HHP and heat treatments. All treated samples (except samples treated at 400 MPa/10 min and 600 MPa/15 min) were positively correlated with PC1, indicating hexanal, *(E)*-2-hexenal, 1-octen-3-ol, 1-octen-3-one and 1-penten-3-one were enhanced after treatments. However, the content of 1-hexanol was decreased by HHP and heat treatments.

GC-MS analysis demonstrated that some of the original volatiles disappeared while some new volatiles appeared after treatments and the content of volatiles changed (Table 3). Alcohols, aldehydes, ketones, esters, and acids were the most numerously represented volatiles classes in kiwifruit pulp beverage. By contrast, the levels of most aldehydes such as hexanal, *(E)*-2-hexenal, nonanal as well as *(E, E)*-2,4-Heptadienal and alcohols such as ethanol and 1-octen-3-ol increased when kiwifruit pulp beverage treated by HHP. In contrast, HHP caused loss in 1-octanol, 1-hexanol and 2-octanol. On the other hand, HHP at 400 MPa/15 min and 500 MPa/5 min retained phenylethyl alcohol while heat-treated samples could not detect this aroma compound. Similarly, the highest 3-octanone content was found in pulp beverage treated at 400 MPa for 10 min while HT caused lowest 3-octanone content. Because complex chemical reactions occurred when pulp beverage treated by HT, which could lead to losses of volatiles in fruit [44]. The content of ketones and acids were low in kiwifruit pulp beverage. However, acetic acid, pentyl ester, acetic acid, and hexyl ester were not detectable in treated samples. Because these volatiles were unstable that was influenced by heat treatment or HHP processing. Ethyl acetate has a pleasant ethereal-fruity and brandy-like aroma [43]. The level of ethyl acetate was not modified significantly after HHP. However, all treated samples were detected a remarkable reduction in the content of butyl acetate and butyl acetate were not detectable when samples treated by pressure greater than 400 MPa. The decrease in the content of butyl acetate after HHP treatment may be due to two reasons: (1) HHP activates or inactivates the enzymes that synthesize esters in fruit; (2) esters are hydrolyzed after HHP treatment [45]. Similarly, Yi et al. [46] also reported the content of esters such as butyl acetate and isobutyl acetate in HHP-treated apple juice were lower than those of untreated juice.

The main volatiles identified in this study were hexanal, *(E)*-2-hexenal, 1-hexanol, 1-octen-3-ol, 1-octen-3-one, 1-penten-3-one, butyl acetate, and ethyl acetate. In addition, hexanal and *(E)*-2-hexenal showed the highest concentrations among all volatiles. They are considered as the most important classes of compounds involved in the perception of volatiles in kiwifruit. HHP and heat treatments resulted in significant (*p* < 0.05) enhancement in the levels of hexanal and *(E)*-2-hexenal, which may be related to autoxidation of lipid in fruit and enzymatic oxidation reactions [47]. Linoleic acid and linolenic acid are converted into hydroperoxides, the 13-hydroperoxide produced is easily converted to C6 aldehyde through cleavage by a hydroperoxide lyase [48]. Moreover, kiwifruit pulp beverage processed at 400 MPa for 5 min showed the highest hexanal content, which was significantly different from heat-treated samples. Porretta et al. [49] also observed an increase in hexanal content of tomato juice after HHP treatment (500 MPa/3 min). In comparison, the higher increase of *(E)*-2-hexenal content was found in pulp beverage treated at 400 MPa for 10 min. The increase in these volatiles may contribute to maintaining freshness of kiwifruit pulp beverage because hexanal imparts grassy notes to kiwifruit and *(E)*-2-hexenal is responsible for green and fruity aroma.

Significant (*p* < 0.05) decrease of 1-hexanol content and increase of 1-octen-3-ol content as well as 1-octen-3-one content were observed after heat and HHP treatments. Additionally, the highest levels of these three compounds were detected in HHP treatment at 400 MPa for 10 min. Previous study also observed a significant reduction in 1-hexanol content as well as an increase in the level of 1-octen-3-ol in HHP-treated (400 MPa for 1 and 150 s) red plum puree with respect to the untreated puree [50]. HHP treatment activated the activity of some glycosidases, so that the glycoside-bound alcohol in kiwifruit pulp beverage was released [51]. Different treatments showed different impacts on 1-penten-3-one. With the exception of HHP treatments (400 MPa for 15 min and 500 MPa for 5–10 min), which had no significant effect on 1-penten-3-one content, the other HHP treatments resulted in significant increase in 1-penten-3-one content. Furthermore, higher 1-penten-3-one content was found in samples processed at 400 MPa for 5 min. 

**Figure 6:**
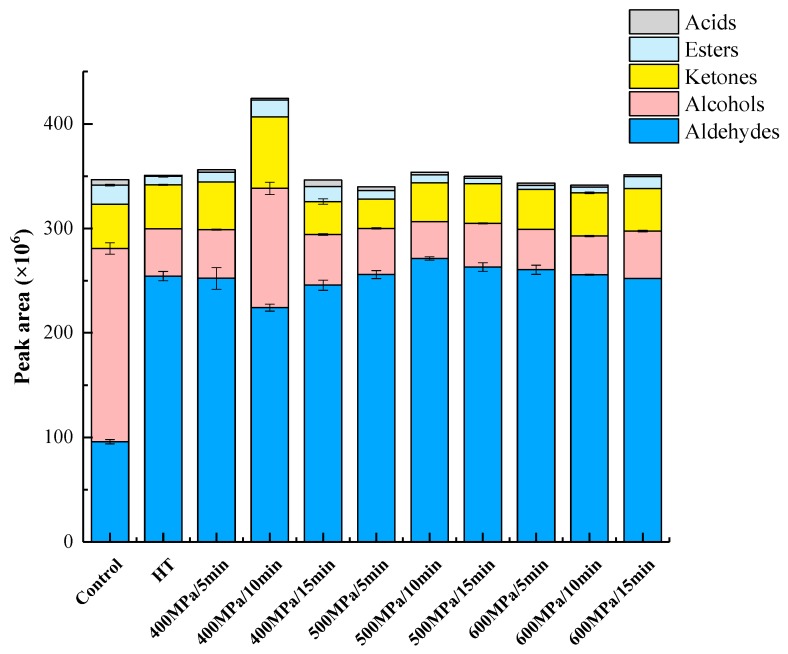
Effect of HHP treatment on volatile groups in kiwifruit pulp beverage on day 0. Control, kiwifruit pulp beverage treated without HT or HHP. HT, heat treatment (85 °C for 10 min). HHP, high hydrostatic pressure (400–600 MPa for 5–15 min). Results were expressed as means ± SD, *n* = 3.

Effect of HHP treatment on volatile groups in kiwifruit pulp beverage was shown in Figure 6. We calculated the sum of peak areas to get the total amount of volatile compounds for a particular group (e.g., alcohols, aldehydes, ketone, esters, and acids). Through the sum we can evaluate the effect of different treatments on volatile compounds for a particular group. Analysis of volatiles showed that aldehydes and alcohols were the most represented classes of compounds in kiwifruit pulp beverage. Aldehydes play an important role in the characteristic aroma of kiwifruit pulp beverage [50]. HHP and heat treatments improved the flavor of pulp beverage since they significantly (*p* < 0.05) enhanced the total aldehydes content. Besides, the highest increase of aldehydes content was observed in samples treated at 500 MPa for 10 min. Similarly, the total content of aldehydes in red plum purée was also slightly increased after HHP treatment [50]. HHP treatments (400 MPa for 5 min/10 min) significantly increased the total ketones content while decrease of ketones concentration was detected in HT and the rest of HHP treatments. By contrast, the total content of alcohols and esters in all treated samples fell significantly, which may be attributed to oxidation of alcohols after treatments, thereby producing aldehydes [52]. Interestingly, this also would be part of the reason for the increase in the aldehydes content of treated samples. These results were similar to previous study about red plum puree and model systems containing fruit esters in buffer solution, in which the total arbitrary area units of alcohols and esters decreased respectively after HHP [45,50]. Among all treatments, the significantly higher alcohols concentration in pulp beverage treated at 400 MPa for 10 min suggested that this treatment could be positive to preserve alcohols in pulp beverage. As for acid compounds, the total content of acids in samples increased by HHP treatment at 400 MPa for 15 min. On the contrary, HT treatments resulted in significant loss in acids. This observation agreed with work of Viljanen et al. [53] who found that thermal treatment decreased content of volatiles. Collectively, 400–500 MPa for 5–15 min resulted in highest levels of main volatiles and volatile groups, suggesting that HHP ranged from 400 to 500 MPa seem to be suitable for preserving the original aroma and enhancing characteristic aroma in kiwifruit pulp beverage.

#### 3.4.2. Changes in Volatiles of HHP-Treated Kiwifruit Pulp Beverage during 40-Day Storage

**Figure 7:**
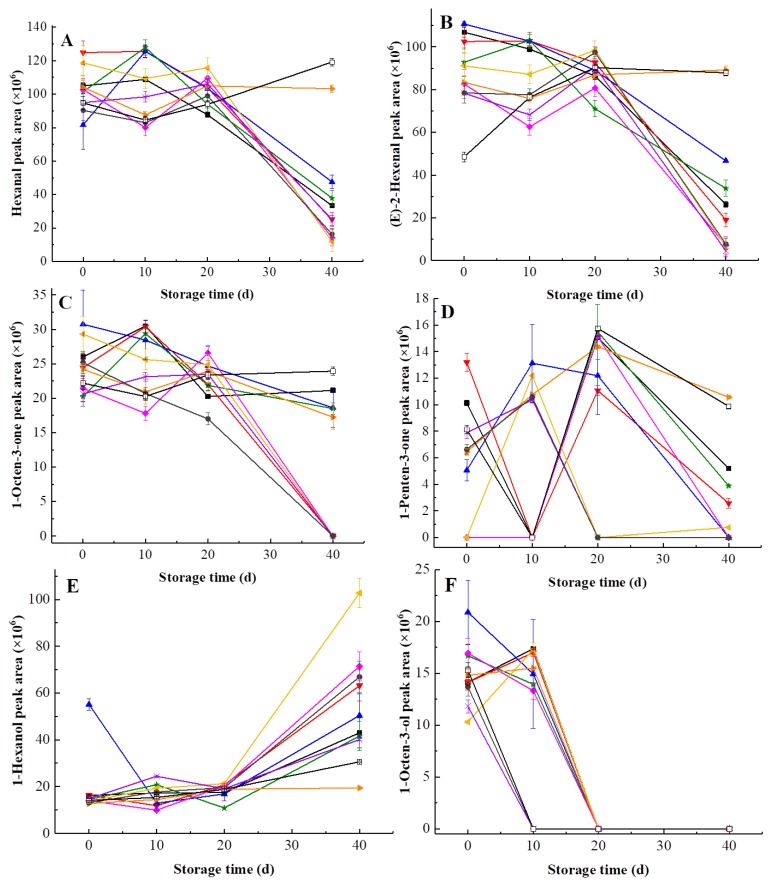
Changes of main volatiles in heat-treated and HHP-treated kiwifruit pulp beverage during storage at 4 °C for 40 days. (**A**) Hexanal content; (**B**) *(E)*-2-hexenal content; (**C**) 1-octen-3-one content; (**D**) 1-penten-3-one; (**E**) 1-hexanol content; (**F**) 1-octen-3-ol content. Heat treatment (85 °C for 10 min) (■), HHP (high hydrostatic pressure, 400-600 MPa for 5-15 min): 400 MPa/5 min (**▼**), 400 MPa/10 min (**▲**), 400 MPa/15 min (**★**), 500 MPa/5 min (**◆**), 500 MPa/10 min (**◀**), 500 MPa/15 min (**▶**), 600 MPa/5 min (**×**), 600 MPa/10 min (**●**), 600 MPa/15 min (□). All data were expressed as means ± SD, *n* = 3.

Main volatiles of kiwifruit pulp beverage were found to change over 40 days (Figure 7). All volatiles in heat-treated and HHP-treated kiwifruit pulp beverage during storage at 4 °C for 40 days were shown in Table S1–S3. HHP treatments at 500 MPa/15 min and 600 MPa/15 min showed better effect of characteristic aroma protection due to slight changes in content of hexanal as well as *(E)*-2-hexenal during storage. Nevertheless, the content of hexanal and *(E)*-2-hexenal in heat-treated and the rest of HHP-treated samples indicated reduction with time. In accordance with this result, Yi et al. [54] reported that the levels of hexanal and *(E)*-2-hexenal in Hayward kiwifruit puree declined with time. Similarly, the 1-octen-3-one content decreased during storage and was not detectable in samples processed at 500 MPa for 5–10 min and 600 MPa for 5-10 min on the 40th day, indicating long term storage resulted in a diminished 1-octen-3-one content. As was shown in Figure 7D, 1-penten-3-one content fluctuated in 40 days while 1-penten-3-one of HHP-treated (500 MPa/15 min) samples was less affected by storage. As a consequence, HHP at 500 MPa for 15 min was positive to preserve 1-penten-3-one in kiwifruit pulp beverage.

As seen in Figure 7E, 1-hexanol content increased over 40 days. In study of Navarro et al. [55], the same trend was observed in 1-hexanol content of strawberry puree processed at 400 MPa for 20 min during 30 days at 4 °C. This may due to the residual activity of lipoxygenase [50]. Differences between the 1-hexanol content in samples of different treatments became larger on the 40th day. Higher content of 1-hexanol was detected in pulp beverage processed at 500 MPa/10 min (16.44%), which was even bigger than initial 1-hexanol content of untreated samples (15.07%). It can be seen in Figure 7F that the 1-octen-3-ol content gradually fell during storage, which was not detectable at all after 20 days. Particularly, 1-octen-3-ol was undetectable in samples treated at 600 MPa on the 10th day, revealing that extremely high pressure caused serious loss in 1-octen-3-ol.

**Figure 8:**
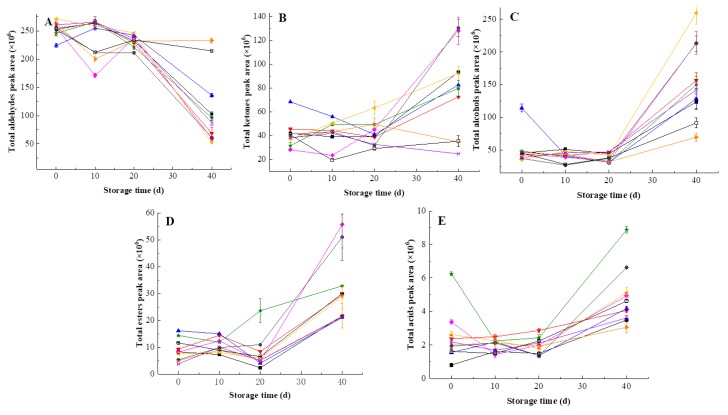
Changes of volatile groups in heat-treated and HHP-treated kiwifruit pulp beverage during storage at 4 °C for 40 days. (**A**) Total aldehydes content; (**B**) total ketones content; (**C**) total alcohols content; (**D**) total esters content; (**E**) total acids content. Heat treatment (85 °C for 10 min) (■), HHP (high hydrostatic pressure, 400–600 MPa for 5–15 min): 400 MPa/5 min (**▼**), 400 MPa/10 min (**▲**), 400 MPa/15 min (**★**), 500 MPa/5 min (**◆**), 500 MPa/10 min (**◀**), 500 MPa/15 min (**▶**), 600 MPa/5 min (**×**), 600 MPa/10 min (**●**), 600 MPa/15 min (□). All data were expressed as means ±SD, *n* = 3.

The total content of aldehydes decreased while the content of the other four volatile groups (alcohols, ketones, esters and acids) increased along the time of storage (Figure 8). Yen and Lin [56] also observed reduction of many aldehyde compounds content after 60-day storage in HHP-treated guava juice. In first 20 days, no difference was found in total aldehydes content of different treated samples. Interestingly, HHP at 500 MPa/15 min and 600 MPa/15 min maintained higher total aldehydes content on the 40th day, indicating that these two treatments were more favorable for the retention of aldehydes compared with HT and the rest of HHP treatments. Figure 8B and Figure 8C clearly showed that the total content of ketones and alcohols in pulp beverage processed at 500 MPa for 10 min increased steadily and remained stable over 20 days, which revealed that HHP at 500 MPa for 10 min was more conducive to the retention of alcohols and ketones than HT. During storage, heat-treated samples showed lower total content of esters and acids than HHP-treated samples. Additionally, the total content of acids in samples processed by 400 MPa for 15 min was higher than those of HT and other HHP treatments at the initial time and on the 40th day. Obviously, HHP showed a greater retention of esters and acids than heat treatment.

Analysis of volatiles in kiwifruit pulp beverage during 40-day storage clearly showed that HHP at 400–500 MPa showed better aroma retention effect when compared with HT. It is generally known that aldehydes such as hexanal and *(E)*-2-hexenal, which are volatiles associated with the odor of grass, leaf and fruit, contribute a lot to the aroma of kiwifruit. By contrast, the content of hexanal and *(E)*-2-hexenal as well as the total aldehydes content in HHP-treated samples (500 MPa/15 min) remained stable at a high value over 40 days, indicating that this treatment is conducive to the retention of important characteristic volatiles in kiwifruit pulp beverage. Furthermore, HHP treatment caused more intense fruity and grassy notes of the kiwifruit pulp beverage.

## 4. Conclusions

It can be concluded that greater retention of vitamin C, color and volatiles were observed in samples processed by HHP than heat-treated samples during storage. Particularly, Hexanal, *(E)*-2-hexenal and aldehydes, which characterized as the major contributor to the ‘fruit, fresh, grassy and green’ notes of kiwifruit, were retained stable after HHP treatment at 500 MPa for 15 min. HHP is possible to be effective and promising as an alternative to traditional heat treatment in juice production industry.

## Figures and Tables

**Table 1:** Changes in pH of heat-treated and high hydrostatic pressure (HHP)-treated kiwifruit pulp beverage during storage at 4 °C for 40 days.

Treatments		Storage Time (Day)			
		0	10	20	40
Control		3.03 ± 0.02 ^b^	3.28 ± 0.01 ^a^	3.29 ± 0.01 ^a^	3.29 ± 0.02 ^a^
HT		3.02 ± 0.02 ^b^	3.28 ± 0.01 ^a^	3.28 ± 0.01 ^a^	3.28 ± 0.00 ^a^
400 MPa	5 min	3.01 ± 0.02 ^b^	3.29 ± 0.01 ^a^	3.28 ± 0.00 ^a^	3.28 ± 0.01 ^a^
	10 min	3.02 ± 0.02 ^b^	3.28 ± 0.00 ^a^	3.27 ± 0.00 ^a^	3.29 ± 0.01 ^a^
	15 min	3.00 ± 0.03 ^b^	3.27 ± 0.02 ^a^	3.28 ± 0.00 ^a^	3.27 ± 0.02 ^a^
500 MPa	5 min	2.99 ± 0.02 ^b^	3.29 ± 0.00 ^a^	3.29 ± 0.02 ^a^	3.28 ± 0.03 ^a^
	10 min	3.01 ± 0.02 ^b^	3.28 ± 0.01 ^a^	3.29 ± 0.00 ^a^	3.27 ± 0.01 ^a^
	15 min	3.02 ± 0.01 ^b^	3.28 ± 0.00 ^a^	3.28 ± 0.01 ^a^	3.29 ± 0.03 ^a^
600 MPa	5 min	2.98 ± 0.02 ^b^	3.28 ± 0.02 ^a^	3.27 ± 0.02 ^a^	3.28 ± 0.01 ^a^
	10 min	3.00 ± 0.01 ^b^	3.28 ± 0.01 ^a^	3.28 ± 0.02 ^a^	3.28 ± 0.02 ^a^
	15 min	3.03 ± 0.02 ^b^	3.28 ± 0.01 ^a^	3.29 ± 0.01 ^a^	3.29 ± 0.02 ^a^

Control, kiwifruit pulp beverage treated without HT or HHP. HT, heat treatment (85 °C for 10 min). HHP, high hydrostatic pressure (400–600 MPa for 5–15 min). Results were expressed as means ± SD, *n* = 3. Different letters indicate the data are different significantly (*p* < 0.05).

**Table 2:** Color of kiwifruit pulp beverage after HHP treatment on day 0.

Treatments		L*	a*	b*	ΔE
Control		75.05 ± 3.93	0.83 ± 0.19 ^a^	12.23 ± 2.25	—
HT		79.34 ± 0.82	−0.04 ± 0.02 ^b^	8.93 ± 0.86	5.48
400 MPa	5 min	75.21 ± 4.70	0.67 ± 0.24 ^a^	11.24 ± 3.78	1.02
	10 min	73.23 ± 9.01	0.81 ± 0.22 ^a^	12.04 ± 4.30	1.83
	15 min	75.21 ± 0.44	0.93 ± 0.12 ^a^	12.26 ± 0.44	0.19
500 MPa	5 min	75.08 ± 4.60	0.65 ± 0.23 ^a^	10.86 ± 2.53	1.38
	10 min	78.32 ± 2.56	0.67 ± 0.33 ^a^	9.35 ± 3.06	4.36
	15 min	73.69 ± 8.52	0.88 ± 0.20 ^a^	11.44 ± 3.68	1.57
600 MPa	5 min	77.65 ± 0.82	0.54 ± 0.11 ^a^	10.99 ± 0.50	2.90
	10 min	78.70 ± 0.75	−0.10 ± 0.01 ^b^	9.09 ± 1.88	4.90
	15 min	79.92 ± 2.15	0.23 ± 0.02 ^a^	8.39 ± 1.80	5.41

Control, kiwifruit pulp beverage treated without HT or HHP. HT, heat treatment (85 °C for 10 min). HHP, high hydrostatic pressure (400–600 MPa for 5–15 min). L*, the brightness value on a black (0) to white (100) scale. a*, hue on a green (−) to red (+) scale. b*, hue on a blue (−) to yellow (+) axis. ∆E, the total color difference between treated samples and untreated samples at the initial time. Results were expressed as means ±SD, *n* = 3. Different letters indicate the data are different significantly (*p* < 0.05). Data without superscripts are not significantly different.

**Table 3:** Changes of volatiles in kiwifruit pulp beverage treated with HHP on day 0.

Volatiles	Peak Area (×10^6^)										
	Control	HT	400 MPa			500 MPa			600 MPa		
			5 min	10 min	15 min	5 min	10 min	15 min	5 min	10 min	15 min
**Aldehydes**											
3-Hexenal	--	0.98 ± 0.12 ^a^	--	--	--	0.67 ± 0.18 ^b^	--	--	0.74 ± 0.12 ^b^	--	0.67 ± 0.25 ^b^
Hexanal	58.68 ± 1.81 ^e^	104.92 ± 2.06 ^b^	124.79 ± 6.87 ^a^	81.67 ± 14.62 ^d^	101.42 ± 7.75 ^bc^	102.73 ± 8.31 ^b^	118.67 ± 10.06 ^a^	104.11 ± 3.8 ^b^	94.98 ± 5.25 ^bc^	90.30 ± 5.94 ^cd^	94.73 ± 3.94 ^bc^
*(E)*-2-Hexenal	32.92 ± 1.81 ^g^	106.87 ± 2.37 ^ab^	102.50 ± 5.37 ^b^	110.74 ± 1.00 ^a^	92.82 ± 6.62 ^c^	82.64 ± 6.87 ^e^	91.13 ± 7.56 ^cd^	83.38 ± 3.00 ^de^	78.33 ± 4.56 ^e^	78.45 ± 4.75 ^e^	48.53 ± 2.25 ^f^
Nonanal	1.38 ± 0.19 ^d^	5.31 ± 1.88 ^abc^	5.13 ± 1.31 ^abc^	4.25 ± 0.69 ^c^	4.50 ± 1.38 ^bc^	6.31 ± 1.88 ^abc^	7.06 ± 0.81 ^ab^	7.19 ± 0.94 ^a^	7.00 ± 1.94 ^ab^	6.88 ± 2.06 ^abc^	7.50 ± 2.56 ^a^
2-Propenal	--	--	--	--	--	--	--	--	--	1.56 ± 0.13 ^a^	1.19 ± 0.06 ^b^
Butanal	--	1.13 ± 0.25 ^a^	1.00 ± 0.13 ^ab^	1.00 ± 0.19 ^ab^	0.75 ± 0.13 ^bc^	0.75 ± 0.19 ^bc^	0.75 ± 0.13 ^bc^	0.63 ± 0.19 ^c^	0.75 ± 0.19 ^bc^	0.69 ± 0.13 ^c^	0.63 ± 0.13 ^c^
2-Butenal	0.63 ± 0.13 ^e^	7.06 ± 0.75 ^bc^	8.06 ± 1.69 ^b^	9.81 ± 1.75 ^a^	6.75 ± 0.81 ^bc^	7.94 ± 1.25 ^b^	5.63 ± 0.81 ^c^	6.06 ± 0.88 ^c^	6.69 ± 0.63 ^bc^	6.06 ± 0.81 ^c^	6.81 ± 0.69 ^bc^
Pentanal	--	8.00 ± 1.25 ^ab^	9.19 ± 2.69 ^a^	4.06 ± 0.94 ^d^	6.88 ± 1.44 ^bc^	7.19 ± 0.94 ^abc^	7.69 ± 1.25 ^abc^	6.00 ± 0.94 ^bcd^	6.63 ± 0.81 ^bc^	5.75 ± 0.75 ^cd^	5.63 ± 0.75 ^cd^
3-methyl-Butanal	0.94 ± 0.31 ^e^	2.13 ± 0.44 ^a^	1.88 ± 0.25 ^ab^	1.94 ± 0.19 ^ab^	1.94 ± 0.04 ^ab^	--	1.56 ± 0.19 ^bcd^	1.25 ± 0.25 ^de^	1.69 ± 0.19 ^bc^	1.56 ± 0.19 ^bcd^	1.44 ± 0.06 ^cd^
*(E)*-2-decenal	--	0.44 ± 0.06 ^de^	0.38 ± 0.06 ^e^	--	--	0.63 ± 0.19 ^cd^	0.63 ± 0.13 ^cd^	1.26 ± 0.21 ^a^	0.81 ± 0.13 ^bc^	1.00 ± 0.25 ^b^	1.00 ± 0.13 ^b^
Octanal	--	2.22 ± 0.37 ^bcd^	2.03 ± 0.23 ^cde^	1.70 ± 0.15 ^e^	1.90 ± 0.20 ^de^	2.49 ± 0.34 ^ab^	2.91 ± 0.25 ^a^	2.55 ± 0.25 ^ab^	2.64 ± 0.22 ^ab^	2.27 ± 0.27 ^bcd^	2.40 ± 0.20 ^bc^
*(E,E)*-2,4-Heptadien-al	--	1.25 ± 0.21 ^c^	1.64 ± 0.24 ^bc^	3.64 ± 0.70 ^a^	1.70 ± 0.23 ^bc^	1.92 ± 0.33 ^b^	1.35 ± 0.20 ^c^	1.38 ± 0.19 ^c^	1.54 ± 0.25 ^bc^	1.23 ± 0.18 ^c^	1.56 ± 0.26 ^bc^
2-decenal	0.60 ± 0.03 ^de^	4.04 ± 1.05 ^c^	2.75 ± 0.77 ^cd^	3.89 ± 0.98 ^c^	4.47 ± 1.37 ^bc^	4.58 ± 1.46 ^bc^	5.01 ± 1.78 ^bc^	--	6.68 ± 1.74 ^ab^	7.48 ± 2.01 ^a^	8.42 ± 2.05 ^a^
2-Undecenal	0.81 ± 0.13 ^d^	4.69 ± 0.31 ^c^	3.44 ± 1.31 ^c^	4.56 ± 0.25 ^c^	5.44 ± 0.21 ^b^	4.81 ± 0.25 ^c^	--	8.38 ± 2.06 ^a^	8.38 ± 1.88 ^a^	9.81 ± 1.56 ^a^	10.25 ± 1.94 ^a^
**Alcohols**											
Ethanol	--	2.81 ± 0.38 ^b^	3.19 ± 0.09 ^a^	1.69 ± 0.31 ^c^	1.38 ± 0.19 ^c^	0.44 ± 0.13 ^d^	0.44 ± 0.19 ^d^	0.31 ± 0.06 ^de^	0.38 ± 0.06 ^d^	0.25 ± 0.19 ^de^	0.25 ± 0.06 ^de^
1-Pentanol	--	--	--	2.44 ± 0.07 ^a^	0.25 ± 0.02 ^c^	0.31 ± 0.06 ^b^	--	--	--	--	--
1-Hexanol	94.19 ± 6.00 ^a^	16.25 ± 0.31 ^cd^	16.38 ± 0.81 ^c^	55.13 ± 2.48 ^b^	14.56 ± 1.00 ^cd^	14.00 ± 1.19 ^cd^	13.81 ± 1.06 ^cd^	12.81 ± 0.44 ^d^	14.88 ± 0.81 ^cd^	13.13 ± 0.81 ^cd^	14.13 ± 0.63 ^cd^
1-Nonanol	--	--	--	--	--	--	--	--	--	--	0.19 ± 0.00
1-Octen-3-ol	--	14.13 ± 0.25 ^bcd^	14.13 ± 0.75 ^bcd^	20.88 ± 3.06 ^a^	16.69 ± 1.06 ^ab^	16.94 ± 1.44 ^ab^	10.31 ± 0.13 ^d^	14.81 ± 0.56 ^bcd^	11.81 ± 0.63 ^cd^	13.69 ± 0.88 ^bcd^	15.31 ± 0.69 ^bc^
1-Octanol	19.31 ± 3.50 ^a^	3.63 ± 0.44 ^c^	3.75 ± 0.19 ^c^	11.50 ± 0.31 ^b^	3.31 ± 0.25 ^c^	3.25 ± 0.56 ^c^	--	3.19 ± 0.38 ^c^	--	--	2.63 ± 0.44 ^c^
2-Octanol	2.94 ± 0.25	--	--	--	--	--	--	--	--	--	--
Phenylethyl Alcohol	7.56 ± 2.06 ^a^	--	--	--	3.38 ± 0.13 ^b^	1.31 ± 0.06 ^c^	--	--	--	--	--
Glycidol	8.06 ± 1.38	--	--	--	--	--	--	--	--	--	--
1-penten-3-ol	--	1.44 ± 0.19 ^b^	--	2.25 ± 0.25 ^a^	--	--	1.31 ± 0.13 ^b^	--	--	--	1.13 ± 0.06 ^c^
3-methyl-1-Butanol	52.88 ± 7.63	--	--	--	--	--	--	--	--	--	--
*(E)*-2-Hexen-1-ol	--	9.31 ± 0.56 ^b^	8.56 ± 1.81 ^bc^	20.38 ± 1.38 ^a^	8.06 ± 0.25 ^bcd^	7.00 ± 0.13 ^d^	7.50 ± 0.36 ^cd^	6.94 ± 0.19 ^d^	8.06 ± 0.31 ^bcd^	6.94 ± 0.36 ^d^	8.00 ± 0.38 ^cd^
**Ketones**											
3-Hexanone	0.19 ± 0.06 ^c^	0.25 ± 0.13 ^bc^	0.50 ± 0.10 ^a^	--	0.50 ± 0.19 ^a^	0.38 ± 0.13 ^ab^	0.44 ± 0.06 ^a^	--	0.44 ± 0.13 ^a^	0.38 ± 0.00 ^ab^	0.50 ± 0.06 ^a^
2-Hexanone	0.56 ± 0.06 ^cd^	1.06 ± 0.13^bc^	2.31 ± 0.25 ^a^	--	2.25 ± 1.88 ^a^	1.63 ± 0.13 ^ab^	1.88 ± 0.19 ^ab^	--	--	1.50 ± 0.13 ^abc^	2.19 ± 0.19 ^a^
2-Pentanone	--	--	--	--	--	0.19 ± 0.02 ^b^	0.31 ± 0.06 ^a^	--	0.25 ± 0.13 ^ab^	--	--
1-Penten-3-one	0.56 ± 0.09 ^e^	10.13 ± 0.19 ^b^	13.19 ± 0.69 ^a^	5.06 ± 0.81 ^d^	--	--	--	6.44 ± 0.25 ^cd^	7.88 ± 0.44 ^bc^	6.63 ± 0.38 ^cd^	8.13 ± 0.31 ^bc^
1-Octen-3-one	9.88 ± 2.91 ^h^	26.00 ± 0.56 ^bc^	24.44 ± 1.56 ^cde^	30.75 ± 4.94 ^a^	20.25 ± 1.44 ^g^	21.44 ± 1.88 ^efg^	29.31 ± 2.63 ^ab^	24.19 ± 0.88 ^cdef^	20.63 ± 1.13 ^fg^	25.19 ± 1.56 ^cd^	22.19 ± 1.00 ^defg^
2,5-Hexanedione	--	1.31 ± 0.25 ^a^	--	--	--	--	--	1.13 ± 0.19 ^b^	--	0.19 ± 0.01 ^c^	0.19 ± 0.02 ^c^
3-Octanone	11.25 ± 1.13 ^b^	--	--	15.63 ± 1.69 ^a^	0.31 ± 0.06 ^d^	--	2.38 ± 0.19 ^c^	--	0.25 ± 0.06 ^d^	0.25 ± 0.13 ^d^	0.31 ± 0.06 ^d^
3-Pentanone	2.63 ± 0.31 ^b^	--	--	13.31 ± 2.00 ^a^	--	--	--	--	--	--	--
3-Heptanone	2.88 ± 0.38 ^b^	1.75 ± 0.13 ^c^	3.50 ± 0.25 ^a^	--	3.44 ± 0.31 ^a^	2.63 ± 0.19 ^b^	--	1.13 ± 0.13 ^d^	--	--	3.31 ± 0.31 ^a^
2-Heptanone	4.75 ± 0.63 ^a^	1.13 ± 0.06 ^de^	1.75 ± 0.19 ^bc^	1.75 ± 0.31 ^bc^	1.50 ± 0.13 ^bcd^	1.31 ± 0.13 ^de^	1.44 ± 0.13^cd^	0.94 ± 0.06 ^e^	1.50 ± 0.19 ^b^	1.44 ± 0.13 ^cd^	1.88 ± 0.25 ^b^
**Esters**											
Ethyl Acetate	5.20 ± 0.50	4.88 ± 0.38	4.94 ± 0.29	4.81 ± 0.56	5.00 ± 0.38	5.13 ± 0.25	5.38 ± 0.25	4.88 ± 0.25	4.81 ± 0.31	4.94 ± 0.13	5.00 ± 0.19
Acetic acid, pentyl ester	2.75 ± 0.81	--	--	--	--	--	--	--	--	--	--
Butyl Acetate	4.38 ± 1.31 ^a^	1.88 ± 0.02 ^b^	2.00 ± 0.06 ^b^	--	--	--	--	--	--	--	--
Acetic acid, hexyl ester	3.56 ± 1.19	--	--	--	--	--	--	--	--	--	--
n-Propyl acetate	--	0.64 ± 0.08 ^abcde^	0.88 ± 0.21 ^ab^	0.61 ± 0.18 ^bcde^	0.91 ± 0.20 ^a^	0.59 ± 0.17 ^cde^	0.71 ± 0.23 ^abcd^	0.41 ± 0.11 ^e^	0.53 ± 0.15 ^cde^	0.49 ± 0.14 ^de^	0.80 ± 0.20 ^abc^
Decanoic acid, ethyl ester	1.19 ± 0.31 ^a^	0.44 ± 0.13 ^cd^	0.44 ± 0.19 ^cd^	0.31 ± 0.13 ^d^	0.63 ± 0.13 ^bc^	0.31 ± 0.06 ^d^	0.81 ± 0.13 ^b^	--	--	--	--
Octanoic acid, ethyl ester	1.44 ± 0.38 ^bc^	0.50 ± 0.06 ^ef^	0.56 ± 0.13 ^ef^	--	0.89 ± 0.22 ^cde^	2.56 ± 0.75 ^a^	1.94 ± 0.75 ^ab^	1.25 ± 0.50 ^cd^	1.13 ± 0.19 ^cde^	0.94 ± 0.13 ^cde^	0.75 ± 0.13 ^de^
**Acids**											
2-ethyl-Hexaoic acid	0.38 ± 0.13 ^ef^	0.31 ± 0.06 ^f^	0.75 ± 0.19 ^b^	0.56 ± 0.06 ^cd^	0.94 ± 0.19 ^a^	0.69 ± 0.19 ^bc^	0.81 ± 0.13 ^ab^	0.50 ± 0.06 ^de^	0.75 ± 0.13 ^b^	0.75 ± 0.25 ^b^	0.81 ± 0.10 ^ab^
Octanoic acid	2.19 ± 1.22 ^b^	0.25 ± 0.00 ^e^	0.44 ± 0.00 ^de^	1.00 ± 0.11 ^cd^	4.19 ± 0.31 ^a^	1.94 ± 0.06 ^b^	1.13 ± 0.06 ^c^	0.88 ± 0.06 ^cde^	0.81 ± 0.06 ^cde^	0.63 ± 0.06 ^cde^	0.50 ± 0.00 ^cde^
Nonanoic acid	0.25 ± 0.01	0.25 ± 0.13	0.38 ± 0.19	--	0.31 ± 0.03	0.38 ± 0.06	0.44 ± 0.19	0.44 ± 0.25	0.44 ± 0.13	0.38 ± 0.00	0.31 ± 0.13
Dodecanoic acid	0.56 ± 0.13	--	--	--	--	--	--	--	--	--	--
n-Decanoic acid	1.50 ± 0.31 ^b^	--	--	--	0.81 ± 0.25 ^c^	0.38 ± 0.13 ^d^	2.00 ± 0.07 ^a^	0.19 ± 0.06 ^de^	0.19 ± 0.00 ^de^	0.19 ± 0.01 ^de^	--

“--” indicated the aroma compound was not detected in samples. Control, kiwifruit pulp beverage treated without HT or HHP. HT, heat treatment (85 °C for 10 min). HHP, high hydrostatic pressure (400–600 MPa for 5–15 min). Results were expressed as means ±SD, *n* = 3. Different letters indicate the data are different significantly (*p* < 0.05). Data without superscripts are not significantly different.

## Supplementary Materials

The following are available online at https://www.mdpi.com/2304-8158/9/4/485/s1, Table S1: Volatiles in kiwifruit pulp beverage treated with HHP after 10-day storage, Table S2: Volatiles in kiwifruit pulp beverage treated with HHP after 20-day storage, Table S3: Volatiles in kiwifruit pulp beverage treated with HHP after 40-day storage.
