# Supplementary material for: Changes in Physicochemical Properties and Volatiles of Kiwifruit Pulp Beverage Treated with High Hydrostatic Pressure
Source: Foods. 2020 Apr 12;9(4):485. doi: 10.3390/foods9040485 (PMC7230769; doi:10.3390/foods9040485)
Supplement: Supplementary file 1 [file foods-09-00485-s001.pdf]

Table S1. Volatiles in kiwifruit pulp beverage treated with HHP after 10-day storage.

**Volatiles**	**Peak area (×10^6)**									
	**HT**	**400 MPa**			**500 MPa**			**600 MPa**		
		**5 min**	**10 min**	**15 min**	**5 min**	**10 min**	**15 min**	**5 min**	**10 min**	**15 min**
Hexanal	108.81±4.19	125.63±3.94	125.56±3.56	127.88±4.50	80.06±4.81	109.31±5.81	87.56±2.19	98.38±3.00	82.94±3.44	84.50±2.25
(E)-2-Hexenal	99.00±3.38	102.81±3.13	102.69±5.13	103.13±3.81	62.63±4.00	87.19±4.38	75.88±1.75	68.19±2.63	77.63±2.75	76.38±1.31
Nonanal	6.00±1.19	5.75±1.88	5.50±1.31	6.63±0.69	4.19±1.38	5.31±1.88	5.06±0.88	4.44±0.94	4.88±1.94	4.25±0.86
2-Propenal	0.25±0.06	--	--	--	--	--	--	0.19±0.03	--	--
Butanal	0.81±0.25	0.88±0.05	0.81±0.14	0.81±0.06	0.88±0.09	0.75±0.03	0.63±0.01	0.63±0.02	0.81±0.02	0.75±0.03
2-Butenal	6.31±1.05	6.19±1.75	4.94±1.29	3.94±1.15	3.31±0.81	5.06±1.25	4.50±0.81	4.50±0.88	4.13±0.63	3.94±0.81
Pentanal	6.88±1.25	7.75±2.69	8.06±0.94	8.25±1.44	5.38±0.94	6.88±1.25	5.94±0.94	5.88±1.19	5.94±0.78	5.56±0.72
3-methyl-Butanal	1.75±0.31	1.94±0.25	1.69±0.19	1.81±0.14	1.44±0.04	1.94±0.19	1.75±0.25	1.69±0.19	1.63±0.19	1.44±0.07
(E)-2-decenal	0.38±0.03	--	--	--	--	--	--	0.25±0.04	0.19±0.02	--
Octanal	18.38±2.37	20.44±2.23	20.13±2.15	22.31±1.20	10.44±1.34	18.31±1.25	14.75±1.22	13.19±1.22	11.19±1.27	13.25±1.20
(E,E)-2,4-Heptadienal	14.19±1.21	16.38±1.24	12.81±1.70	12.69±1.23	10.81±0.33	17.38±1.20	13.94±0.19	11.13±0.85	16.75±1.26	10.50±0.96
2-decenal	20.63±2.03	9.31±1.05	10.81±1.77	14.50±0.98	8.50±0.87	10.19±1.46	9.38±1.18	14.75±1.04	8.31±1.01	9.44±1.05
2-Undecenal	3.06±0.13	1.00±0.31	1.44±0.31	1.88±0.25	1.13±0.21	1.31±0.25	1.25±0.26	2.31±0.88	1.06±0.16	1.38±0.56
Ethanol	0.50±0.09	0.75±0.10	0.56±0.08	0.44±0.05	0.25±0.02	0.38±0.03	0.38±0.04	0.31±0.02	0.31±0.01	0.31±0.03
1-Pentanol	0.38±0.05	--	--	--	0.38±0.07	0.38±0.02	0.31±0.01	0.31±0.02	--	0.19±0.01
1-Hexanol	17.31±1.39	11.81±0.38	12.94±3.00	20.81±0.06	9.94±0.02	19.31±0.63	14.50±0.13	24.44±1.50	17.75±0.50	15.38±0.38
1-Octen-3-ol	17.38±0.13	17.00±0.38	14.94±5.25	13.94±0.06	13.31±0.81	17.31±0.63	15.50±0.13	--	--	--
1-Octanol	3.56±0.99	--	--	3.25±0.76	--	--	2.50±0.53	2.25±0.46	--	--
1-penten-3-ol	--	1.94±0.19	1.69±0.25	1.56±0.13	1.19±0.36	1.69±0.19	1.44±0.31	1.25±0.36	--	1.19±0.21
(E)-2-Hexen-1-ol	9.25±1.81	10.25±1.51	10.94±1.36	11.69±1.31	8.06±1.42	9.81±1.38	7.75±1.31	12.44±1.57	9.25±1.41	8.44±1.21

3-Hexanone	0.25±0.03	--	0.56±0.06	0.69±0.05	0.25±0.03	0.56±0.05	0.63±0.04	0.38±0.02	--	0.56±0.05
2-Hexanone	--	2.44±0.25	2.50±0.16	--	--	1.75±0.13	2.13±0.25	1.50±0.19	--	--
1-Penten-3-one	--	--	13.13±2.94	--	--	12.19±0.13	10.75±0.25	10.38±0.19	10.56±0.22	--
1-Octen-3-one	30.50±1.31	30.38±1.88	28.44±2.88	29.38±0.81	17.81±1.06	25.63±1.50	20.94±1.75	23.13±0.63	20.69±1.88	20.25±1.56
2,5-Hexanedione	--	0.19±0.02	0.25±0.03	--	0.13±0.02	0.06±0.00	0.06±0.01	--	--	--
3-Octanone	--	--	--	--	--	--	0.25±0.03	--	--	--
3-Heptanone	1.19±0.13	3.69±0.38	3.88±0.31	4.00±0.71	--	3.50±0.76	3.06±0.69	2.19±0.51	3.44±0.81	2.75±0.38
2-Heptanone	1.19±0.21	2.63±0.31	2.50±0.63	2.56±0.19	--	2.19±0.25	1.94±0.15	2.00±0.31	2.13±0.22	1.81±0.19
Ethyl Acetate	5.50±0.50	7.31±1.38	6.81±1.38	8.19±0.85	5.63±0.95	6.38±0.31	5.06±0.37	7.25±0.51	8.88±0.46	6.06±0.31
Butyl Acetate	1.31±0.31	--	1.75±0.14	2.19±0.13	1.63±0.24	1.94±0.10	2.31±0.34	2.19±0.37	2.13±0.24	1.63±0.14
n-Propyl acetate	3.13±0.38	5.25±0.51	5.69±0.48	6.81±0.90	3.38±0.27	3.00±0.53	5.06±0.51	5.06±0.65	3.31±0.34	4.88±0.90
2-ethyl-Hexaoic acid	0.69±0.08	0.94±0.10	0.94±0.09	1.00±0.05	0.44±0.03	0.75±0.06	0.63±0.12	0.50±0.13	0.81±0.14	--
Octanoic acid	0.50±0.08	1.13±0.08	0.75±0.06	0.69±0.02	0.50±0.03	0.94±0.08	0.75±0.06	0.50±0.05	0.75±0.09	0.56±0.04
Nonanoic acid	0.44±0.16	0.44±0.13	0.50±0.03	0.56±0.13	0.38±0.05	0.56±0.06	0.63±0.12	0.69±0.11	0.56±0.06	0.94±0.15
n-Decanoic acid	--	--	--	--	0.13±0.01	--	0.13±0.00	--	--	--

"--" indicated the aroma compound was not detected in samples. HT, heat treatment (85°C for 10 min). HHP, high hydrostatic pressure (400-600 MPa for 5-15 min). Results were expressed as means ± SD, n = 3.

Table S2. Volatiles in kiwifruit pulp beverage treated with HHP after 20-day storage.

**Volatiles**	**Peak area (×10⁶)**									
	**HT**	**400 MPa**			**500 MPa**			**600 MPa**		
		**5 min**	**10 min**	**15 min**	**5 min**	**10 min**	**15 min**	**5 min**	**10 min**	**15 min**
Hexanal	87.63±1.23	103.81±3.74	103.31±3.66	94.00±4.30	109.56±4.51	115.75±5.31	104.94±2.09	106.13±2.98	98.94±3.31	93.88±2.19
(E)-2-Hexenal	86.38±1.36	92.63±3.13	89.00±3.00	71.06±3.51	80.69±4.05	98.44±4.38	86.88±1.72	90.69±2.60	97.38±2.75	90.31±1.32
Nonanal	4.31±0.18	4.69±0.81	5.00±0.94	4.88±0.88	4.81±0.99	4.88±0.878	4.56±1.08	4.31±0.98	4.19±1.00	4.13±1.02
Butanal	0.81±0.12	0.75±0.12	0.63±0.19	0.88±0.17	0.75±0.10	0.81±0.11	0.94±0.22	0.75±0.12	1.38±0.23	0.75±0.12
2-Butenal	7.44±1.69	5.06±0.95	6.69±1.13	7.38±1.19	6.13±1.06	5.13±0.94	5.94±0.82	6.44±1.04	4.31±0.74	7.0±1.07
Pentanal	6.56±1.25	7.00±0.94	6.38±1.44	7.63±0.96	7.31±0.88	7.69±1.21	7.31±0.94	7.19±0.75	6.94±1.19	6.88±0.75
3-methyl-Butanal	2.13±0.31	1.69±0.44	1.50±0.15	1.88±0.19	1.88±0.04	1.88±0.19	1.94±0.25	1.75±0.19	1.50±0.06	1.94±0.13
Octanal	11.88±0.75	14.94±1.13	15.50±1.75	12.69±1.19	14.81±1.13	14.88±1.08	13.50±0.93	15.25±1.23	11.19±1.25	11.75±0.75
(E,E)-2,4-Heptadienal	29.19±2.21	14.63±1.24	22.44±1.70	25.44±1.23	23.00±1.33	19.31±1.20	18.69±0.99	21.44±1.25	11.25±1.18	22.25±1.26
2-decenal	7.25±0.93	9.31±1.05	8.50±0.77	5.63±0.98	7.19±0.98	6.81 ±1.46	6.38±1.78	6.44±1.74	5.00±1.05	5.88±1.34
2-Undecenal	0.88±0.13	1.13±0.31	0.94±0.25	0.56±0.11	0.81±0.25	0.56±0.06	0.69±0.16	0.63±0.21	0.44±0.10	0.63±0.11
Ethanol	0.38±0.06	0.38±0.03	0.25±0.01	0.44±0.02	0.31±0.01	0.31±0.02	0.25±0.00	0.31±0.02	0.31±0.06	0.25±0.02
1-Pentanol	0.56±0.07	0.44±0.02	0.44±0.03	0.50±0.07	0.44±0.03	0.50±0.05	0.44±0.04	0.50±0.02	0.50±0.03	0.50±0.05
1-Hexanol	17.50±0.13	20.75±0.38	16.88±3.00	10.88±0.06	20.31±0.02	21.31±0.63	18.94±0.13	19.13±0.50	19.56±0.50	18.88±0.38
1-Octanol	2.19±0.67	2.75±0.44	2.38±0.31	--	1.31±0.11	1.31±0.10	1.19±0.27	--	--	--
1-penten-3-ol	2.00±0.31	1.44±0.22	1.63±0.19	2.06±0.25	--	1.75±0.38	1.69±0.18	1.75±0.27	--	1.94±0.28
(E)-2-Hexen-1-ol	8.69±0.56	11.00±1.81	8.63±1.38	8.31±0.95	10.25±1.13	11.75±1.36	9.56±0.79	10.38±0.31	10.50±0.96	10.25±0.78
3-Hexanone	--	0.50±0.06	0.25±0.03	0.63±0.02	0.25±0.01	0.63±0.03	0.25±0.00	0.31±0.02	0.69±0.02	0.50±0.03
2-Hexanone	0.75±0.06	--	--	1.50±0.16	--	2.75±0.38	0.94±0.05	--	2.38±0.35	--
1-Penten-3-one	15.06±1.13	11.06±1.38	12.19±2.94	15.50±2.06	15.13±2.13	--	14.38±1.13	--	--	15.75±1.19

1-Octen-3-one	20.25±1.31	22.00±1.88	24.69±2.88	21.88±1.81	26.56±1.06	24.88±1.50	23.94±1.75	23.50±0.63	17.00±1.88	23.38±1.56
2,5-Hexanedione	--	--	--	--	0.06±0.01	0.13±0.02	--	--	--	--
3-Octanone	--	0.25±0.03	--	0.25±0.02	--	--	--	--	0.25±0.01	--
3-Heptanone	1.06±0.16	--	1.13±0.24	--	1.25±0.06	3.81±0.93	1.25±0.13	--	3.06±0.25	2.81±0.19
2-Heptanone	1.50±0.29	--	1.44±0.38	--	1.63±0.22	2.13±0.19	1.44±0.15	--	1.81±0.32	1.69±0.16
Ethyl Acetate	--	3.31±1.19	2.50±0.83	--	2.56±0.72	--	2.56±0.33	2.63±0.54	3.81±0.92	3.25±0.88
Acetic acid, pentyl ester	1.13±0.06	1.69±0.19	--	1.56±0.31	1.19±0.13	1.75±0.13	1.13±0.19	1.25±0.17	1.75±0.09	1.56±0.25
Butyl Acetate	1.13±0.31	1.69±0.25	--	1.56±0.14	1.19±0.33	1.75±0.18	1.13±0.12	1.25±0.24	1.75±0.31	1.56±0.25
n-Propyl acetate	2.25±0.78	5.38±0.91	2.69±0.38	2.31±0.50	3.13±0.67	5.75±0.53	3.44±0.71	3.56±0.65	6.69±1.14	4.38±0.90
2-ethyl-Hexaoic acid	0.69±0.08	1.06±0.17	--	0.75±0.09	0.56±0.04	--	0.56±0.06	0.63±0.03	--	0.75±0.07
Octanoic acid	0.50±0.08	1.06±0.22	1.06±0.13	1.13±0.19	1.13±0.22	1.25±0.11	1.06±0.21	1.19±0.19	0.69±0.06	1.19±0.13
Nonanoic acid	0.31±0.07	--	0.31±0.02	0.56±0.02	0.31±0.03	0.56±0.05	0.31±0.04	0.31±0.03	0.69±0.07	0.31±0.07

"--" indicated the aroma compound was not detected in samples. HT, heat treatment (85°C for 10 min). HHP, high hydrostatic pressure (400-600 MPa for 5-15 min). Results were expressed as means ± SD, n = 3.

Table S3. Volatiles in kiwifruit pulp beverage treated with HHP after 40 day-storage.

**Volatiles**	**Peak area (×10⁶)**									
	**HT**	**400 MPa**			**500 MPa**			**600 MPa**		
		**5 min**	**10 min**	**15 min**	**5 min**	**10 min**	**15 min**	**5 min**	**10 min**	**15 min**
3-Hexenal	--	--	--	--	--	--	--	0.38±0.07	--	--
Hexanal	33.31±1.17	25.06±2.90	47.56±3.54	37.81±4.50	14.13±1.79	11.81±1.81	103.25±2.15	24.56±2.01	16.25±1.44	119.19±2.25
(E)-2-Hexenal	26.31±1.40	19.00±3.13	46.69±2.13	33.81±3.83	7.13±4.03	6.56±4.35	89.19±1.75	4.50±2.63	7.75±2.77	87.88±1.30
Nonanal	4.25±0.19	2.94±0.88	4.19±0.31	4.50±0.69	3.13±0.38	2.88±0.88	4.81±0.81	5.19±0.94	5.19±1.06	6.00±1.25
Butanal	0.69±0.15	0.75±0.13	1.13±0.19	0.56±0.13	0.69±0.19	0.38±0.13	0.75±0.19	0.69±0.09	0.25±0.05	0.75±0.08
2-Butenal	5.94±1.75	4.06±0.69	4.88±0.75	4.75±0.81	2.13±0.94	3.38±0.81	6.25±0.95	1.69±0.13	2.38±0.71	--
Pentanal	--	1.81±0.25	3.06±0.69	--	--	--	6.19±0.94	--	--	6.63±1.13
3-methyl-Butanal	2.63±0.31	1.44±0.44	1.56±0.25	1.75±0.39	11.81±0.84	3.25±0.89	1.81±0.25	3.06±0.79	6.19±1.19	1.88±0.27
(E)-2-decenal	--	--	--	--	--	--	--	0.44±0.09	--	--
Octanal	25.44±1.37	7.69±1.23	12.38±1.15	10.69±1.04	--	13.19±1.20	12.69±1.33	13.19±1.19	--	20.25±2.25
(E,E)-2,4-Heptadienal	5.44±0.81	--	7.69±1.70	7.44±1.37	--	--	21.25±2.20	--	--	11.00±1.27
2-decenal	9.56±1.03	6.31±0.95	14.56±0.77	9.69±0.83	9.06±0.33	8.94±0.46	--	23.25±1.78	13.88±0.74	--
2-Undecenal	1.06±0.13	0.63±0.21	1.38±0.31	1.13±0.25	0.75±0.21	1.13±0.25	1.31±0.06	1.75±0.88	1.25±0.56	1.00±0.56
Ethanol	2.50±0.38	1.75±0.09	1.31±0.31	1.50±0.19	7.75±1.13	2.75±0.39	1.50±0.16	2.75±0.36	3.75±0.19	2.00±0.63
1-Pentanol	7.56±1.31	5.69±0.81	2.44±0.48	4.13±0.33	14.25±2.48	10.31±1.19	1.75±1.06	4.31±0.81	12.06±1.81	3.13±0.63
1-Hexanol	43.00±6.38	63.19±6.63	50.44±9.19	41.69±6.31	71.44±6.25	102.75±6.25	19.44±0.13	40.13±0.50	66.94±6.75	30.63±0.38
1-Octanol	10.13±1.50	8.63±0.44	8.50±0.89	11.44±1.31	16.19±1.25	14.81±1.56	4.81±0.38	20.19±2.56	17.63±1.88	8.31±0.69
1-penten-3-ol	--	2.19±0.19	1.63±0.25	2.00±0.13	--	3.13±0.73	1.88±0.38	--	--	1.88±0.25
3-methyl-1-Butanol	--	--	1.19±0.33	--	--	--	--	--	--	--
(E)-2-Hexen-1-ol	37.13±2.56	36.75±2.81	30.00±1.38	38.31±1.25	50.94±2.13	60.19±2.36	14.19±1.19	25.94±1.31	50.38±1.36	23.00±1.38

3-Hexanone	0.44±0.06	0.88±0.13	0.44±0.10	1.00±0.19	1.38±0.13	1.13±0.06	--	0.50±0.13	2.00±0.26	--
2-Hexanone	1.31±0.06	2.75±0.33	1.38±0.25	3.25±0.88	4.13±0.93	3.56±0.79	1.38±0.43	1.31±0.19	6.94±1.81	1.44±0.09
2-Pentanone	--	0.63±0.10	--	--	0.81±0.08	--	--	1.00±0.06	--	--
1-Penten-3-one	5.19±0.14	2.56±0.38	--	3.88±0.06	--	0.75±0.13	10.56 ±1.19	--	--	9.88±0.20
1-Octen-3-one	21.13±1.31	--	18.63±1.06	18.50±1.50	--	--	17.25±1.42	--	--	23.94±1.75
2,5-Hexanedione	--	--	--	--	--	--	--	0.38±0.09	--	--
3-Octanone	22.88±1.13	18.56±1.69	16.69±1.06	20.75±1.19	24.69±2.06	24.94±2.13	--	--	6.81±0.89	9.88±1.00
3-Pentanone	16.81±1.13	12.38±1.49	7.81±0.75	11.81±1.00	21.88±1.19	23.88±1.06	--	5.31±0.31	21.69±1.19	4.50±0.69
3-Heptanone	3.44±0.38	5.38±0.13	2.88±0.25	6.19±0.31	9.31±1.19	8.25±1.13	--	--	10.25±1.53	--
2-Heptanone	3.13±0.63	3.38±0.76	2.69±0.19	3.56±0.31	5.69±1.13	4.75±0.83	--	--	8.69±1.77	--
Ethyl Acetate	9.75±0.50	18.19±1.38	13.75±0.99	8.69±0.56	12.56±1.38	9.44±0.85	8.56±1.25	3.31±0.31	11.75±0.73	6.63±0.69
Butyl Acetate	1.25±0.31	1.88±0.02	1.19±0.26	1.94±0.29	2.75±0.38	2.31±0.31	1.31±0.25	--	2.44±0.19	1.38±0.13
n-Propyl acetate	3.81±0.58	7.63±1.21	4.88±0.18	7.13±1.20	8.69±1.17	9.63±1.23	4.00±0.11	3.44±0.15	8.38±1.14	4.75±0.20
2-ethyl-Hexaoic acid	0.94±0.13	1.13±0.26	0.94±0.19	1.25±0.16	1.44±0.19	1.44±0.19	0.88±0.13	0.88±0.11	1.94±0.33	1.06±0.21
Octanoic acid	1.13±0.22	1.38±0.11	1.69±0.31	2.06±0.76	1.19±0.24	2.13±0.13	1.19±0.25	1.00±0.06	2.56±0.96	1.44±0.33
Nonanoic acid	0.38±0.01	0.31±0.13	0.44±0.19	0.56±0.03	0.25±0.06	0.31±0.09	0.44±0.05	0.56±0.13	0.38±0.10	0.69±0.13

"--" indicated the aroma compound was not detected in samples. HT, heat treatment (85°C for 10 min). HHP, high hydrostatic pressure (400-600 MPa for 5-15 min). Results were expressed as means ± SD, n = 3.
